# Leveraging monitoring, evaluation, and learning to scale the Enabling Inclusion^®^ program for children with disabilities in India and globally

**DOI:** 10.3389/fpubh.2023.1165034

**Published:** 2023-12-12

**Authors:** Marie Brien, Franzina Coutinho, Dinesh Krishna, Lotte van der Haar, Joost de Laat, Sankara Raman Srinivasan, Navamani Venkatachalapathy

**Affiliations:** ^1^Enabling Inclusion Program, Amar Seva Sangam, Tenkasi, Tamil Nadu, India; ^2^Department of Family and Community Medicine, University of Toronto, Toronto, ON, Canada; ^3^Utrecht Centre for Global Challenges, Utrecht School of Economics, Utrecht University, Utrecht, Netherlands

**Keywords:** early childhood development, child disability, LMIC, IT supported early interventions, monitoring, evaluation, and learning (MEL), scaling

## Abstract

**Introduction:**

Children with disabilities in low- and middle-income countries face many challenges and lack adequate services, including access to rehabilitation professionals. To address this lack of access, Amar Seva Sangam Ayikudy (ASSA), a non-governmental organization (NGO) in India, designed a technology-leveraged rehabilitation program called Enabling Inclusion^®^ (EI^®^), and implemented it in one state (Tamil Nadu, India) before scaling it. The model is supported by the EI^®^ app, which enables organizations to screen, assess and monitor progress of children with disabilities via rehabilitation specialists and community rehabilitation workers, and to provide family-centered, goal-based interventions. An extensive monitoring, evaluation, and learning (MEL) framework is embedded into the program. This paper explores how this MEL system supported the scaling of the EI^®^ model, reaching additional beneficiaries nationally and globally.

**Methods:**

This paper describes ASSA’s MEL framework and demonstrates its use for decision-making in the process of scaling. It also explores how collaborations with various government departments, NGOs, and private partners contributed to the scaling of the EI^®^ model and technology.

**Results:**

Scaling of the EI^®^ program was achieved by (1) expansion of the program in rural Tamil Nadu (vertical scale-up) in partnership with the Tamil Nadu government and private partners, and (2) by licensing the EI^®^ app and model to other NGOs in various states in India and globally (horizontal scale-up). Systematic examination of key program and performance indicators, as well as stakeholder feedback, informed decisions to modify the EI^®^ app over time. This included further customizing to the needs of children and service providers, covering a greater range of age groups and contexts, and modifying service delivery models. Child functional independence, participation, and inclusion was further strengthened by mobilizing parent empowerment groups, community awareness programs, school advocacy, and entitlements from the government. Flexibility in the implementation model of the EI^®^ app allowed for adaptation to local contexts and organizations, and facilitated its scale-up.

**Conclusion:**

A dynamic, inclusive, and locally grounded MEL system, a flexible and collaborative approach, and an adaptive implementation model increased the accessibility of an early intervention and childhood rehabilitation program for children with disabilities and their families throughout the state of Tamil Nadu, across India, and internationally.

## Introduction

The protection of children with disabilities is enshrined in the UN Convention on the Rights of the Child (1), the Convention on the Rights of Persons with Disabilities (UNCRPD) (2), and the Sustainable Development Goals (SDGs) (3). The SDG target 4.2 requires that “all girls and boys have access to quality early childhood development, care, and pre-primary education so that they are ready for primary education,” and SDG 3 seeks to “ensure healthy lives and promote well-being for all at all ages” (3, 4). The global commitment to move toward Universal Health Coverage (UHC) is one step in this direction, but the challenge is significant. Children with disabilities include those with long-term physical, mental, intellectual, or sensory impairments. Globally, nearly 240 million children living with disabilities are unable to realize basic rights, including access to early stimulation and nurturing care, and face stigma, discrimination, and exclusion from communities and schools (4), which limit their participation in society on an equal basis with their non-disabled peers (5–7). The challenges for children with disabilities in low- and middle-income countries (LMICs) are particularly pronounced, as low-resource settings early in life further predisposes children to adverse health and compromised developmental outcomes, yet children in LMICs also have the least access to public and private support services. Addressing those challenges requires early intervention and rehabilitation, as the early years are critical for child development and school readiness (8, 9), and requires a family and community participatory approach.

There is a growing body of evidence on the effectiveness of interventions for early childhood development; however, there is scant information on how to implement effective programs at scale (9, 10). Early childhood development (ECD) programs may be scaled to new groups in similar settings and to different populations in new settings (11). As the context changes with scaling, many factors that ultimately impact a program’s effectiveness will also change, including the socio-economic, cultural, and political context, as well as features of the implementing organizations and their partners (e.g., financial resources, social relations, and leadership) (12–14). Therefore, understanding the new contexts where scaling will happen is essential for scaling to be effective (15).

Monitoring, evaluation, and learning (MEL) systems can support organizations in understanding these new contexts and inform adaptation and delivery strategies (9). This paper explores how a responsive MEL system supported the scaling of the Enabling Inclusion^®^ (EI) app and model by Amar Seva Sangam (ASSA), a non-governmental organization (NGO) in South India. This paper is a follow-up to Krishna et al. (16), which described the findings from a rapid-cycle evaluation of ASSA’s early intervention program for children with developmental disabilities in South India.

### Scaling contexts for Amar Seva Sangam’s Enabling Inclusion^®^ program

There are many barriers to the equitable provision of early intervention services in India. Research suggests that, until 2016, only 10% of the approximately 2.3 million children under the age of 6 with disabilities in India were accurately diagnosed, and even fewer were receiving appropriate rehabilitation (17, 18). The lack of public community-based child rehab programs hinders timely screening, diagnosis, and appropriate intervention (19, 20). Barriers include low awareness and high disability stigma, lack of rehabilitation centres and trained specialists, especially in rural or semi-urban areas, long distances, lack of transportation to urban centres, high costs, and long waiting times to urban rehab centres (21). Many of these barriers can be addressed by mobilizing community support and harnessing the power of connectivity through technology.

In this context, ASSA designed and implemented a technology-leveraged program and software application called the Enabling Inclusion^®^ (EI^®^). The EI^®^ app is designed to support rehabilitation specialists and community rehabilitation workers to screen and assess, and monitor progress of children with developmental disabilities, especially in rural areas, and to provide child rehab service providers (e.g., therapists, community rehab workers, NGO management, and caretakers) with a menu of evidence-based, family-centered interventions. The specific modules in the EI^®^ app are: Child developmental screening, Rehabilitation and Environmental Assessments and Evaluations, Family-Centred Goal Setting and Intervention/Therapy Planning, Intervention Assignment, Scheduling, Monitoring, Awareness and Training Programs, and Dashboard and Reporting. Impact evaluation studies by Krishna et al. (16) and Muthukaruppan et al. (19) report a positive impact of the program, including improved service provider work satisfaction, improved program engagement, and school enrollment, decreased caregiver strain, and increased parent empowerment.

Given its potential to reach vulnerable children with disabilities and the findings on positive impacts, ASSA decided to try to scale the EI^®^ app and model to new localities across India and globally. Its strategy to scale used a combination of 2 approaches: vertical and horizontal scale-up. According to UNICEF (22), vertical scale-up involves the expansion of an existing program or set of programs, where the benefit, value, or duration of the program is increased for some or all current recipients, new components are added, or new beneficiaries within the existing geography are increased (22, 23). Horizontal scale-up, on the other hand, refers to an expansion of an existing program, or set of programs, to increase beneficiaries from new geographies and communities (22).

Over its 40-year history, ASSA activities have primarily focused on sustaining and growing its activities through private grant funding in one district (Tenkasi District) of Tamil Nadu. To facilitate vertical scale-up within Tamil Nadu, ASSA cultivated partnerships with the Tamil Nadu government to increase beneficiaries and community awareness and bring greater financial sustainability. Meanwhile, ASSA’s approach to horizontal scale-up focused on licensing the EI^®^ app and model to other NGOs in various states in India and abroad, as well as forming knowledge partnerships with government departments.

## Methods

### The MEL framework for the Enabling Inclusion^®^ program

To support the process of scaling the EI^®^ program, ASSA developed a Monitoring, evaluation, and learning (MEL) system based on the ‘5 aspirations of Measurement for Change’ described by Krapels et al. (24), namely for the MEL system to be dynamic, inclusive, informative, interactive, and people-centered (24, 25). The MEL framework supports monitoring and decision-making at different stages of the program, from initial design through implementation and scaling, with ongoing adaptations of the MEL system and the program. In this section, we describe five features of the MEL framework: (1) a new organizational structure, (2) key program indicators, (3) key performance indicators, (4) stakeholder feedback, and (5) how MEL was used for decision-making.

#### A new organizational structure to support scaling

To support the scaling process and ensure quality, ASSA established a new organizational structure, building on its 40 years of experience providing child rehabilitation services in rural South Tamil Nadu. This new structure included a social enterprise and a centre of excellence.

The social enterprise, *Amar Seva Global Association* (ASGA) was established in 2020 to create awareness about the EI^®^ program and establish new partners. ASGA is a non-profit company with the legal authority to license the use of the EI^®^ app to other organizations globally. The primary objective of ASGA is to enable access to early intervention and rehabilitation services for children with disabilities globally through technology-based rehab solutions. The secondary objective is to generate revenue to fund the Amar Seva Centre of Excellence, allowing for greater financial sustainability for the centre.

A key component of ASSA’s MEL framework is the *Amar Seva Centre of Excellence for Rehabilitation and Development of Children with Disabilities (ASCE),* which was established in 2021 to support the organization and partner organizations in the scaling process and ensure the quality of the innovation is maintained. The centre’s vision is to develop an inclusive world where children, families, and communities are provided the resources that allow all children with disabilities to reach their full potential. The centre supports a collaborative ecosystem of stakeholders, including national-, state-, and local-government, NGOs, private sector, parents, children, and service providers. Its activities include partnership development, supporting MEL and scientific research activities, training of partners, co-creating solutions and ongoing technology development.

#### Key program indicators

As part of its implementation, the EI^®^ program tracks a comprehensive set of indicators through the EI^®^ app. It utilizes a standardized process flow and internationally validated tools for child development and parent outcome indicators [for details, see Krishna et al. (16)]. The app’s data retrieval, reporting, and dashboard features provide important information to support decision making for all program stakeholders.

The program indicators collected include:

- Program staff and stakeholders using the app, including the number and type of service providers (i.e., program managers, rehab therapists, community workers, etc.).
- Demographic and socio-economic data of children and parents reached by the program, such as age and gender, socio-economic indicators (parental education and income), and home, family, community, and school environments.
- Child developmental screening information, including the type of screening instrument used and the impairments and disability diagnosis categories.
- The type of EI^®^ intervention provided, such as physiotherapy, special education, speech, language, and communication therapy, and subclassifications under each type.
- Changes over time in validated child development scores in various domains of development, caregiver strain, and empowerment.
- Participant characteristics in the awareness programs and trainings, including the number, age, and gender of participants attending the sessions.
- Access by children to services, including therapy, education, assistive technology, and government benefits.

For further details, please see Supplementary Table S1.

#### Key performance indicators

The EI^®^ app also produces key performance indicators by program sites, which include the percentage of completed service provider activities (therapy and non-therapy) and utilization rates of various EI^®^ app modules. For further details, please see Supplementary Table S2.

Tracking these program and performance indicators allows the program implementers to understand who is being reached, by whom, with which services, and how active beneficiaries and other stakeholders (e.g., community members) are participating. It also allows for comparisons across program sites/implementation models, nationally and internationally. Some examples of the questions that can be answered using the EI^®^ app are:

- What is the prevalence of children with delayed development from child screening programs?
- What are the types of impairment identified and the medical disability diagnosis of these children?
- What is the program adherence rate (achieved therapy visits divided by planned visits)?
- What are the development progression and trajectories of children receiving therapy in gross motor, self-care, mobility, language and communication, cognitive, social, personal, occupational, recreational, and academic areas of development?
- Do children have improved school enrolment, access to assistive technology, and social/government benefits?
- Do caregivers have a reduction in strain/stress, an increase in family empowerment, and improved interactions with their child?
- Do people in the community who attended awareness programs have improved knowledge, attitudes, and practices regarding childhood disabilities?

To improve outcomes, all EI^®^ program teams are encouraged to reflect on the information generated by the app and set up rapid-cycle action and iterative program change on both an individual child, family, and programmatic level.

#### Stakeholder feedback

In addition to collecting specific indicators through the EI^®^ app, ASSA also systematically gathers feedback from a variety of stakeholders, both through the app itself and outside the app. Using qualitative and quantitative methods feedback from NGO program managers, funders, service providers, beneficiaries, public sector policymakers, and the community in obtained. For example, feedback from children, caregivers, as well as service providers and program management, occurs through surveys built into the EI^®^ app that have to be completed at regular intervals. Surveys are also administered to community members attending awareness programs. Formal focus groups and interviews are conducted regularly with all stakeholders. This feedback plays a critical role in the re-design and upgrades of the EI^®^ app, and in rapid cycle action and iterative program changes more generally.

#### Integrating the information into a MEL framework for decision-making

Combined, the different components of the MEL system collect information across the results chain, from key inputs to key indicators and intermediate outcomes, including stakeholder feedback. To ensure this wealth of information is actually used to inform decisions, ASSA follows a structured approach that governs and guides its use for specific users. Specifically, the organization’s MEL framework is broken down by use for: operational-, supervisory-, and management staff, and by its Board of Directors. This structured approach supports program staff and partners at all levels and roles to ensure the program is reaching those children that are targeted, with high quality of implementation, and make adaptations where necessary in the scaling process required in a specific context. The MEL structure guides decision-making on adaptations of the design of the EI^®^ program, get buy-in from stakeholders, where and whom to reach, and how to ensure program effectiveness in new contexts with different partners.

## Results

### Scaling the EI^®^ program

The Enabling Inclusion^®^ innovation was launched by ASSA in 2014 with direct implementation of the program through private funders in one District of Tamil Nadu. It has grown since, both vertically in Tamil Nadu, and horizontally to new states and abroad. These are highlighted in Table 1.

**Table 1:** Amar Seva Sangam’s Enabling Inclusion^®^ scaling journey.

Vertical scale-up	Horizontal scale-up
Private partnerships (launched 2014) Initial pilot program-Tamil Nadu 1 District	Licensed NGO partnerships (launched 2021) 2 countries (India, Ethiopia) 7 Indian states, 35 Districts 13 NGOs
Tamil Nadu disability department (launched 2020) 2 Districts	Tamil Nadu department of education (launched 2022) 1 District with planned scale-up to all 38 districts in the state
Tamil Nadu department of health (launched 2021) 1 District early intervention centre	

The EI^®^ model’s vertical scale-up involved the expansion of the program within the state of Tamil Nadu, supported by additional private funding and through funding and implementation agreements with two government departments (Disability Department in 2020 and Health Department in 2021). As part of the expansion, ASSA hires and trains service providers to expand the implementation of EI^®^ programs in Tamil Nadu, thus reaching more children and families.

In March 2021, ASSA began expanding the program throughout India and abroad by licensing the EI^®^ app and model to multiple Non-Governmental Organizations (NGOs). As of August 15th, 2023, the program has been adopted by 10 NGOs across five states in India (Tamil Nadu, West Bengal, Uttar Pradesh, Pondicherry, Assam, and Karnataka) and 3 institutions working in the Amhara region in Ethiopia. In addition to licensing to NGOs, horizontal scale-up was achieved through a partnership with the Tamil Nadu Department of Education consisting of a pilot project in one new district and a knowledge partnership to implement a state-wide solution to scaling early intervention and child rehabilitation services.

From initiating the EI^®^ innovation in 2014 until the launching of ASGA and ASCE in 2020, the EI^®^ program’s MEL data found that a total of 55,729 children had been screened for delayed development, 1,136 children have been positively impacted with improved development and function, 1854 caregivers have been positively impacted by decreasing their strain and increasing their empowerment, and 43,056 community members attending awareness programs had improved knowledge, attitudes and practices with regards to child development and disability. The new scaling strategy launched in 2020 resulted in greater outputs. From the initiation of the program till August 15th, 2023, 234,289 children have been screened for delayed development, 8,294 children have been positively impacted with improved development and function through rehab services, 25,684 caregivers have decreased strain and increased empowerment, and 184,356 community members attending awareness programs have improved knowledge, attitudes and practices with regards to child development and disability.

Tables 2–5 show the demographic features of the children and families reached through the 4 scale-up programs (note: the Disability and Health Dept. partnership data are reported together as they are operated in the same geographical area and by the same management team and fall under vertical scale-up).

**Table 2:** Child impairment distribution in the EI^®^ program scaling partnerships.

	Tamil Nadu health and disability dept. partnerships (*N* = 2,973)	Private partnerships (*N* = 3,152)	Licensed NGO partnerships (*N* = 1,102)	Tamil Nadu education dept. partnership (*N* = 1,070)
Impairments	*n* (%)	*n* (%)	*n* (%)	*n* (%)
Physical	1,110 (37.3)	1,207 (38.3)	396 (36.0)	490 (45.8)
Cognition	415 (14.0)	1,061 (33.7)	294 (26.7)	398 (37.2)
Speech	874 (29.4)	495 (15.7)	192 (17.5)	35 (3.3)
Hearing	116 (3.9)	145 (4.6)	30 (2.7)	57 (5.0)
Behavioural	144 (4.8)	128 (4.1)	53 (4.8)	36 (3.4)
Vision	61 (2.1)	42 (1.3)	18 (1.6)	53 (5.0)
Other	253 (8.5)	74 (2.8)	117 (10.5)	1 (0.1)

**Table 3:** Child diagnosis distribution in the EI^®^ program scaling partnerships.

	Tamil Nadu health and disability dept. partnerships (*N* = 2,973)	Private partnerships (*N* = 3,152)	Licensed NGO partnerships (*N* = 1,102)	Tamil Nadu education dept. partnership (*N* = 1,070)
Diagnosis	*n* (%)	*n* (%)	*n* (%)	*n* (%)
ADHD	95 (3.2)	73 (2.3)	48 (4.4)	6 (0.6)
Autism	197 (6.6)	245 (7.8)	156 (14.2)	52 (4.9)
Blindness	16 (0.5)	5 (0.2)	11 (1.0)	7 (0.7)
Cerebral palsy	679 (22.8)	865 (27.4)	245 (22.2)	201 (18.8)
Developmental delay	32 (1.1)	20 (0.6)	45 (4.1)	0 (0)
Down syndrome	119 (4.0)	93 (3.0)	47 (4.3)	26 (2.4)
Dwarfism	4 (0.1)	1 (0)	1 (0.1)	5 (0.5)
Hearing impairment	133 (4.5)	160 (5.1)	39 (3.5)	67 (6.3)
High risk baby	0 (0)	0 (0)	51 (4.6)	0 (0)
Intellectual disability	219 (7.4)	656 (20.8)	164 (14.9)	380 (35.5)
Low vision	43 (1.4)	95 (3.0)	8 (0.7)	39 (3.6)
Mental illness	4 (0.1)	34 (1.1)	6 (0.5)	0 (0)
Multiple disability	119 (4.0)	56 (1.8)	34 (3.1)	122 (11.4)
Muscular dystrophy	26 (0.9)	51 (1.6)	2 (2.0)	24 (2.2)
Other	225 (7.6)	38 (1.2)	48 (4.4)	26 (2.4)
Specific orthopedic Disability	187 (6.3)	125 (4.0)	73 (6.6)	98 (9.2)
Specific learning disability	51 (1.7)	166 (5.3)	12 (1.1)	3 (3.3)
Speech and language disorder	687 (23.1)	443 (14.1)	107 (9.7)	9 (0.8)
Spina bifida	18 (0.6)	25 (0.8)	3 (0.3)	1 (0.4)
Spinal cord injury	2 (0.1)	1 (0)	2 (0.2)	1 (0.1)
NA	117 (3.9)	0 (0)	0 (0)	0 (0)

**Table 4:** Age and gender distributions of children followed by EI^®^ program scaling partnerships.

	Tamil Nadu health and disability dept. partnerships (*N* = 2,973)	Private partnerships (*N* = 3,152)	Licensed NGO partnerships (*N* = 1,102)	Tamil Nadu education dept. partnership (*N* = 1,070)
Age range* (years)	*n* (%)	*n* %	*n* %	*n* %
0–2	553 (18.6)	95 (3.0)	162 (14.7)	13 (1.2)
3–5	1,034 (34.8)	522 (16.6)	357 (32.5)	53 (5)
6–8	931 (31.3)	727 (23.1)	271 (24.6)	236 (22.1)
9–12	439 (14.8)	925 (29.3)	175 (15.9)	421 (39.3)
13–18	14 (0.5)	699 (22.2)	126 (11.5)	340 (31.8)
19–21	0 (0)	93 (3.0)	7 (0.6)	7 (0.7)
>21	0 (0)	91 (2.9)	2 (0.2)	0 (0)
Gender				
Female	1,169 (39.3)	1,194 (37.9)	396 (36.0)	468 (43.7)
Male	1804 (60.7)	1958 (62.1)	704 (64.0)	602 (56.3)

*Age on August 15, 2023.

**Table 5:** Family demographics of children followed by EI^®^ Program scaling partnerships.

	Tamil Nadu health and disability dept. partnerships (*N* = 2,973)	Private partnerships (*N* = 3,152)	Licensed NGO partnerships (*N* = 1,102)	Tamil Nadu education dept. partnership (*N* = 1,070)
Family income level (Rs/Year)*	*n* (%)	*n* (%)	*n* (%)	*n* (%)
0–15,999	129 (4.3)	312 (9.9)	216 (26.5)	27 (2.5)
16,000–49,999	430 (14.5)	379 (12.0)	108 (13.3)	220 (20.5)
50,000 – 300,000	2,367 (79.6)	2,409 (76.4)	437 (53.7)	670 (62.6)
>300,000	47 (1.6)	52 (1.6)	53 (6.5)	15 (1.4)
NA	0 (0)	0 (0)	286 (26.0)	139 (13)
Father’s education				
Illiterate	163 (5.5)	361 (11.5)	76 (8.9)	NA
Anganwadi	13 (1.0)	41 (1.3)	0 (0)	
1-12th standard	2,254 (75.0)	2,240 (71.1)	498 (58.0)	
Diploma	222 (7.5)	204 (6.5)	63 (7.3)	
Undergraduate	221 (7.4)	208 (6.8)	156 (18.2)	
Postgraduate	100 (3.4)	98 (3.1)	65 (7.6)	
Mother’s education				
Illiterate	128 (4.3)	522 (5.6)	86 (10.0)	NA
Anganwadi	8 (1.0)	38 (1.2)	3 (0.3)	
1–12th Standard	2,186 (73.0)	2072 (65.7)	559 (65.2)	
Diploma	165 (5.5)	251 (8.0)	33 (3.8)	
Undergraduate	318 (10.7)	149 (4.7)	117 (13.6)	
Postgraduate	168 (5.7)	522 (16.6)	60 (7.0)	

*India household income distribution: 0–15,999 (below poverty line)16,000–49,999 (low income), 50,000–300,000 (middle class), >300,000 (high income), source: PRICE’s ICE 360 pan-India surveys https://www.ice360.in/ice360/. NA = data not available.

### The EI^®^ program’s MEL framework

In the next section, we present how ASSA’s MEL framework contributed to the vertical and horizontal scale-up process.

#### MEL and vertical scaling

Vertical scaling involved collaborations with two Tamil Nadu government departments, and private partnerships.

##### Scaling with the department of welfare of the differently abled

ASSA’s first vertical scale-up venture involved a partnership with Tamil Nadu’s Department for Welfare of Differently Abled, also known as the Disability Department. The partnership was achieved by lobbying and highlighting the positive results of the pilot study using the EI^®^ app and model in one district (Tenkasi) (16, 19). This department then decided to fund ASSA to scale up the program to two entire new neighbouring districts in Tamil Nadu – Tirunelveli and Tuticorin, through direct implementation with ASSA-hired service providers.

Based on the success of this two-district scale-up venture, underpinned by the information generated by the program’s MEL system, the Tamil Nadu Disability Department launched a statewide program called the RIGHTS project. Funded with the support of the World Bank, it aims to strengthen the social protection systems and capability of the State of Tamil Nadu to promote inclusion, accessibility, and opportunities for persons with disabilities (26). The program has expanded on ASSA’s initial focus of early intervention and child rehab to cover people with disabilities of all ages.

A key component of the RIGHTS project is the development of a centralized registry of children and adults with disabilities. All children in the registry must be provided a diagnosis and a unique disability ID (UDID) card in order to access government maintenance grants, health insurance, transportation support, reasonable accommodations in schools, as well as future rehab services to be provided as part of the RIGHTs project (26). One of the MEL’s key performance indicators, access to social/government benefits, revealed that only 18% of children in the 2 district scale-up program had obtained UDID cards in April 2021. ASSA subsequently conducted stakeholder consultation through a survey of parents, which revealed that 85% of parents either did not know what benefits would be derived from obtaining this card and/or did not know how to apply for it, and 40% cited that having a UDID card would lead to their child being negatively labeled. These MEL findings informed the decision to conduct a campaign to educate parents on the benefits of obtaining a UDID card, how to apply for a UDID card, and to de-stigmatize the process. Through these various efforts, the percentage of children with UDID cards improved to 48% by April 2022.

Based on the success of this rapid cycle action, the Tamil Nadu government is launching a similar campaign to have children with disabilities register for UDID cards state-wide prior to launching the RIGHTs program. In addition, they are in negotiation with ASSA to use the Enabling Inclusion^®^ app solution for the state-wide scale-up program.

##### Scaling with the department of health-Tamil Nadu

Each district in Tamil Nadu has a District Early Intervention Centre (DEIC) operated by the Department of Health, where children under the age of 6 at risk for disabilities can be referred for assessments, further referral, and rehabilitation. DEICs are located in the District Government Hospital and have pediatricians and rehabilitation specialists hired by the State Health Department. Though nearly 600–700 children are assessed at these centres annually, only approximately 30 children (<5%) are able to come to the centres regularly to receive therapy services.

As part of its MEL efforts, ASSA conducted stakeholder consultation through interviews with the Tamil Nadu Department of Health, staff at DEICs, and parents of children seen at DEICs. The consultations highlighted the need for an integration of services with the EI^®^ home-based model. Therefore, the MEL component of the EI^®^ app was upgraded to include a module that would capture additional data required by DEIC staff and a mechanism to facilitate further referral and follow-up and the EI^®^ software solution.

The EI^®^ app was piloted in one (Tenkasi) DEIC starting in February 2021. Prior to the integration of the EI^®^ app, there were no referrals of children from the Tenkasi DEIC to any home-based program. From February 2021 to July 2023, 1836 children were assessed by DEIC staff using the EI^®^ app, of which 659 (36%) were referred to ASSA’s home-based early intervention program. This pilot program demonstrates how NGO and Government services can be integrated and can act as a model for scale-up throughout Tamil Nadu and other states in India.

##### Scaling with private partnerships

Through partnerships with corporate funders and funding agencies, ASSA has directly implemented the EI^®^ app in the Tenkasi district since 2014.^1^ This private funding has sustained different scale-up programs, including a home and centre-based early intervention program, school-age child rehab program, and a special needs school. The conception of each of these programs was guided by the MEL-generated stakeholder feedback, including feedback from funders, service providers, and beneficiaries.

For example, a recent ASSA MEL study has shown that caregivers enrolled in the EI^®^ program report experiencing reduced strain and increased family empowerment (19). Another ASSA’s MEL study highlighted that parents who reported greater strain and less empowerment were also those parents who had fewer peer connections and community supports. Stakeholder feedback indicated the need for greater parent-peer support, community integration, social inclusion, and the ability to advocate for their children (16). Rapid cycle action was taken to form parent empowerment groups within the home-based early intervention program to respond to these parents’ needs. These groups consist of 14 to 18 parents of children with developmental disabilities living in similar geographical areas. They are led by parent leaders and supported by community rehabilitation workers (CRWs) and rehab specialists providing care for those children. The monthly group meetings aim to promote peer connection among parents, gain knowledge on disability rights and resources, and promote child and family community inclusion and parental advocacy. Parent social media groups via WhatsApp were also formed to supplement these parent groups.

A study examining the outcomes of the EI^®^ Program parent groups^2^ and parent social media groups^3^ has been conducted and is pending publication. The study reveals the benefit of these groups in promoting peer support and community integration. Based on these results, ASSA will seek to incorporate strategies to encourage peer group support, community integration, social inclusion, and the ability to advocate for their children as crucial elements of its scaling strategy.

#### MEL and horizontal scaling

The following section describes how the MEL framework supported the horizontal scaling of the EI^®^ program to other states in India and abroad.

##### Scaling with NGO partnerships

ASSA facilitated a significant horizontal scale-up, by licensing the EI^®^ app and model to other NGOs, and providing training, technology implementation, onboarding, and support services. Its integrated MEL activities played a crucial roll in the process.

The initial design for scaling with partner NGOs included an EI^®^ program training using a 5–7 day Train-the-Trainer workshop. The goal of the workshop was to have project leaders train their own service providers in the use of the EI^®^ software and model. ASSA’s MEL-generated data revealed improved outputs and outcomes for the children and families reached via horizontal scaling, including more children with disabilities reached, improved child development, and increased parent empowerment. Despite the improved program indicators, two key elements of the MEL framework, stakeholder feedback, and key performance indicators, revealed gaps in the scaling efforts. Formal feedback indicated that the app’s users (service providers) did not fully understand the app’s various modules and how to use the modules. Feedback also revealed that the app’s design, initially conceptualized for a home-based model, had challenges when implemented in school- or centre-based programs. In addition, many NGOs had school-age children, which they wanted to be covered within the model and app, while the EI^®^ app modules initially covered preschool children only till age 6.

Partner NGO feedback was supported by analyzing the completion rate of the various EI^®^ app module activities across the various program sites. Detailed analysis of this indicator showed that some modules in the EI^®^ app were not being used at all or minimally used by partner NGOs. Feedback from partner service providers revealed that some of the assessments and modules were too time-consuming to complete within their organization’s workflow, particularly for school- or centre-based services.

Several salient rapid-cycle action changes were implemented to respond to feedback from NGO partners. ASSA enhanced the EI^®^ app training program, revamped support services, and upgraded the EI^®^ app. For example, realizing that the train-the-trainer model was inadequate, ASSA started offering direct in-person training of service providers at the partner organization location, allowing ASSA trainers to fully understand the local context and adapt the training accordingly. Furthermore, the EI^®^ app was upgraded to render it fully customizable to the needs of each partner NGO. For example, NGOs now have the flexibility of customizing existing assessments, changing assessments to locally used tools, and creating reports tailored to their needs. The EI^®^ app was also upgraded to cover intervention protocols from birth to 25 years of age, providing children with disabilities a continuous follow-up from birth to early adulthood within the EI^®^ model. Finally, a 24 h technology help desk was set-up to respond to issues faced by users of the EI^®^ app and to fulfill customization requests.

Following these changes, the number of children reported to be enrolled and impacted has increased (a key program indicator), the completion rate of the modules has increased significantly (a key performance indicator), and feedback from NGOs revealed that the EI^®^ app use was better understood, as well as more culturally and contextually appropriate to their setting (stakeholder feedback). ASSA intends to continue its horizontal scaling through NGO licensing and partnerships, using its MEL framework to facilitate program and technology adaptations.

##### Scaling with the department of education-Tamil Nadu

Samagra Shiksha (SS) is the Indian central government program to promote the education of all children (including minorities, socially disadvantaged, and children with disabilities) in Tamil Nadu and is operated by the Tamil Nadu Education Department. Through public sector advocacy that relied significantly on the findings from the MEL-generated evaluation studies, ASSA established a line of communication with Tamil Nadu’s Education Department, presented the EI^®^ solution, and held more in-depth discussions. This networking led to a formal partnership between this Department and ASSA. As part of this agreement, workers in the SS program, including special educators and physiotherapists, integrated the use of the EI^®^ app as part of their regular child rehab and education services across two early intervention centres, ten-block resource centres, 452 government schools, and 561 government-aided schools and their home-based programs in 1 district.

ASSA instituted the MEL framework, gathering stakeholder feedback through meetings with Department of Education policymakers, children, and parents of children receiving services with the program. ASSA recognized that the SS program is established with special educators as the key educational and therapy services provider for children with disabilities, while physiotherapists provide consultative services. Essential changes to the original EI^®^ app were deemed necessary as it was developed for a multidisciplinary approach involving community rehabilitation workers and a team of rehab specialists (physiotherapists, special educators, speech trainers/ therapists, and occupational therapists). The MEL-generated feedback from the SS Program indicated that there would not be enough rehabilitation providers to conduct all the assessments, evaluations, and intervention planning for the various domains of development contained within the EI^®^ app.

To address this concern, ASSA designed a new version of the EI^®^ app with a transdisciplinary approach that integrates therapeutic disciplines for a holistic developmental perspective of the child. The Functional Independence Measure for Children (WeeFIM) (27) was added to the EI^®^ app to facilitate this new transdisciplinary approach. The WeeFIM, a comprehensive validated measure, evaluates all functional aspects of child development (27). ASSA developed corresponding interventions, goals, and strategies within the EI^®^ app, covering all developmental domains that special educators address as part of each child’s individualized education plan. Parents are empowered to learn these interventions and integrate therapeutic strategies into a child’s daily life.

The Department of Education used the EI^®^ app as a pilot in one district to facilitate their learning. However, their scaling goal will involve the creation of their own app that is owned, operated, managed, and supported by the Education Department. While ASSA initially intended for a state-wide scale-up of the EI^®^ app, it quickly realized that in order to scale the impact of its innovation, it needed to be flexible in its approach. Sharing the learnings and co-creating solutions could result in a more significant impact. Therefore, as part of the agreement with the Education Department, ASSA has agreed to be a knowledge partner in the department’s inclusive education strategy via participation in their project management unit (PMU). This PMU is tasked with creating an inclusive education app and a system to scale early intervention, child rehab, and integrated inclusive education services to children with disabilities across the entire state of Tamil Nadu. ASSA will use the pilot in the Tenkasi district with SS workers and learnings from other scale-up programs to co-create this solution as knowledge partners with the Tamil Nadu Education Department. This knowledge partnership will lead to significant scaling in the horizontal scale-up vector.

### Scaling and children being reached

In the process of scaling, the distribution of children being reached by the program has varied across implementation sites. For example, there is variation in the age of children reached as different EI^®^ programs targeted different age groups. Another variation is seen in terms of the demographic features of families enrolled in the NGO partnerships compared to the private and government partnerships (Disability/Health department and Education department). NGO partnerships reach more families below the poverty line (26% compared to 3–10%), as well as lower mother and father education levels. This can be explained by the fact that the state of Tamil Nadu, where private and government partnerships were implemented, has higher average income and education levels, as compared to the other five Indian states where NGO partnerships were implemented (28).

Across all programs, the percentage of boys receiving intervention (ranging from 56 to 62%) exceeded girls. Our findings revealed that more boys were receiving interventions as compared to girls. This is, in part, following the overall sex ratio in India which is skewed towards males. Data also suggests that the prevalence of childhood disabilities is higher in boys as opposed to girls (29). Furthermore, various studies report that access to healthcare services is not equitable among boys and girls (across the life cycle). This inequity often stems from patriarchal practices within families and could potentially also be reflected in our findings (30).

The MEL framework also indicates that the EI^®^ programs may not be reaching families in the lowest income level. While the majority of Indian households are low-income (31), the majority of households reached across the 4 categories of the EI^®^ programs were middle class (54%–79%). While equitable accessibility is at the heart of the EI^®^ model including providing services free of cost to families, the data shows that despite the EI program efforts, lower-income families may have less access to child rehabilitation services. This could be due to economic factors (time spent engaged in therapy means time away from income-earning opportunities) or lack of awareness of the benefits of therapy. Strengthening initiatives to address families’ socio-economic status through livelihood, self-help groups and parent empowerment group may be needed in parallel to providing child rehab services to reach more low income families.

Finally, we can also use ASSA’s MEL data to compare the distribution of children and families reached by the program to (inter)national data on children with impairment and disabilities. While the Global Burden of Disease Study revealed that the most common impairment identified among the pediatric population globally is visual impairment, followed by hearing impairments, intellectual disability, and autism spectrum disorder (10), across all the different EI^®^ programs in India, the most common primary impairment was physical impairment followed by cognitive impairment. Though vision and hearing interventions are in the app, there is more emphasis more on physical and cognitive intervention in the app. A strengthening of vision and hearing intervention within the app and model could allow for a greater reach of children with vision and hearing impairments.

## Discussion

This paper describes how MEL contributed to a sustainable multidimensional scaling of ASSA EI^®^ program, which leverages technology to provide developmental services to children with disabilities and their families. The scale-up involved the expansion of EI^®^ implementation in rural Tamil Nadu (vertical scale-up) in collaboration with the Tamil Nadu government and other funders, as well as licensing of the EI^®^ app and model to other NGOs throughout India and internationally and through knowledge partnership with a government department (horizontal scale-up) (32). In the process, ASSA sought to achieve impacts at scale in terms of human resources, accountability, responsibility, collaborative engagement with all stakeholders, and scope (33, 34). These scale-up efforts were guided by an extensive MEL system involving continuous examination of key program inputs, outputs, activities, program and performance indicators, outcomes, and extensive stakeholder feedback, and acting on the learnings generated through rapid cycle responses (35).

### The MEL experience and the 5 measurement for change aspirations

ASSA’s MEL system has been critical to achieving the sustainable multidimensional impact at scale of the Enabling Inclusion^®^ (EI) Model and App. This section describes how the EI^®^ program’s MEL framework embodies the “*5 aspirations for Measurement for Change*”, described by Krapels et al. (24), that aim for a MEL system to be dynamic, inclusive, informative, interactive, and people centered.

The EI^®^ program’s MEL system’s *dynamic* approach allowed the flexibility and adaptability of design and implementation strategies, including making the EI^®^ app customizable, introducing a transdisciplinary model, and expanding coverage of services to children of all ages. These adaptations were driven by an *informative* MEL system, including key program and performance indicators and stakeholder feedback, each providing complementary data points to guide rapid cycle actions and decision-making.

The MEL system’s *inclusive* approach resulted in an ongoing family-centered approach and stakeholder consultation with families of children with disabilities, leading to the formation of parent groups and initiatives to increase access to government benefits such as disability cards, grants, and services. The MEL system’s *interactive* approach allowed ASSA to observe, track, and utilize interactions with children, families, and service providers and responses while honoring and respecting relationships, resulting in parent empowerment groups and parent social media groups. This *inclusive* and *interactive* approach and the resulting initiatives will be critical to the future scale-up implementation strategy.

The MEL system’s *people-centered* approach allowed successful scaling by measuring whether families with different circumstances and characteristics have been included in the program. Scaling within Tamil Nadu was a successful outcome following government consultations, collaborative meetings, and agreements, leading to funding commitments and knowledge partnerships that will be critical to multidimensional scaling. NGO scaling focused on extensive consultation with and feedback from NGOs; this led to program modifications, including revised training, strengthened support services, and an upgraded, customizable app to make the model more contextually relevant. This *people-centered* approach was essential as each NGO and government has a critical understanding of the unique context of the communities they work in and direct working relationships with people in those communities.

All five measurements for change aspirations (*dynamic, informative, inclusive, interactive, and people-centered*) led to adaptations to the EI^®^ Model to address varied contexts, including different service delivery agents (e.g., health, rehabilitation, and educational workers in various settings), different types of users (governments, NGOs), different modes of service delivery (centre-based, home-based, mobile clinic, school-based), different target groups (e.g., various children age groups- pre-school, school-age to adolescence), and differences in socio-economic and cultural settings.

The multifunctional EI^®^ app underwent ‘adaptations for scaling and because of scaling’. Fidelity to the original design was maintained and focused on the impact outcomes in improved child development and caregiver well-being and access to inclusive education. Flexibility in the implementation model solidified scale-up as the family-centered EI^®^ app was developed to be adaptable to local contexts and organizations while remaining an efficient child development and rehab solution (36, 37).

### Recommendations and conclusion

Monitoring, evaluation, and learning is at the centre of the EI^®^ program, supporting accountability, buy-in, and informing decision making at all levels, and throughout program design, implementation, and adaptation to new contexts. The adaptive implementation framework of the EI^®^ program, guided by the flexible MEL model, was crucial for achieving both vertical scaling in the state of Tamil Nadu, and horizontal scaling to new States in India and abroad. In the process of scaling, children from different ages, living in different contexts, and with different needs and resources, were reached by different types of service providers. The MEL generated information allowed the EI^®^ program to be adapted to these different contexts. ASSA’s MEL approach, guided by the *5 aspirations of Measurement for Change*, provides lessons that may be beneficial to scaling early interventions in other contexts.

## Data availability statement

The data analyzed in this study is subject to the following licenses/restrictions: data is available upon request. Requests to access these datasets should be directed to MB, researchassociate@amarseva.org.

## Author contributions

DK, SS, FC, MB, LH, JL, and NV contributed to the conception and design of all components of the article. DK, SS, MB, and NV contributed to the design of the MEL system and facilitated the rapid cycle evaluation actions described in his paper. MB and FC conducted a literature review and the first draft of the manuscript. DK, LH, and JL wrote sections of the manuscript. All authors contributed to the article and approved the submitted version.

## Footnotes

^1^These funders include Grand Challenges Canada, Handi-care Intl, Kal and Viji Raman Foundation, Azim Premji Foundation, Standard Chartered Bank, Voltas, and UTI Bank.

^2^Proctor K, Patel M, Krishna D, Venkatachalapathy N, Brien M, Langlois S. A capacity-building intervention for parents of children with disabilities in rural South India. (2023) (under review).

^3^Coutinho F, Ogourtsova T, Saxena G, Krishna K, Brien M, Venkatachalapathy N. A case series of tele-rehab health coaching interventions in rural India. (2023) (under review).

## References

[1] United Nations general assembly, convention on the rights of the child, 20 November (1989), United Nations, treaty series, 1577, p. 3. Available at: https://www.unicef.org/child-rights-convention 12344587

[2] United Nations General Assembly, Convention on the rights of persons with disabilities: resolution / adopted by the general assembly, 24 January (2007), A/RES/61/106. Available at: https://www.ohchr.org/en/instruments-mechanisms/instruments/convention-rights-persons-disabilities

[3] United Nations. Sustainable development goals. United Nations, New York, NY (2015). Available at: https://sustainabledevelopment.un.org/

[4] UNICEF. Nearly 240 million children with disabilities around the world, UNICEF’s most comprehensive statistical analysis finds [Press Release]. 2021 Nov 9. Available at: https://www.unicef.org/press-releases/nearly-240-million-children-disabilities-around-world-unicefs-most-comprehensive

[5] World Health Organization. United Nations Children's fund, World Bank Group. Nurturing Care for Early Childhood Development: A framework for helping children survive and thrive to transform health and human potential. Geneva: World Health Organization (2018). Licence: CC BY-NC-SA.;3. Available at: http://apps.who.int/iris/bitstream/handle/10665/272603/9789241514064-eng.pdf?ua=1

[6] Daelmans B Darmstadt GL Lombardi J Black MM Britto PR Lye S. Lancet early childhood development series steering committee. Early childhood development: the foundation of sustainable development. Lancet. (2017) 389:9–11. doi: 10.1016/S0140-6736(16)31659-2 27717607

[7] Gove A Black MM. Measurement of early childhood development and learning under the sustainable development goals. J Hum Dev Capabil. (2016) 17:599–605. doi: 10.1080/19452829.2016.1243520

[8] UNICEF The State of the World's Children (2013). Children with disabilities: From exclusion to inclusion. New York. Available at: https://www.unicef.org/reports/state-worlds-children-2013

[9] Britto PR Singh M Dua T Kaur R Yousafzai AK. What implementation evidence matters: scaling-up nurturing interventions that promote early childhood development. Ann N Y Acad Sci. (2018) 1419:5–16. doi: 10.1111/nyas.13720, PMID: 29791739

[10] Global Research on Developmental Disabilities Collaborators. Accelerating progress on early childhood development for children under 5 years with disabilities by 2030. Lancet Glob Health. (2022) 10:e438–44. doi: 10.1016/S2214-109X(21)00488-5, PMID: 35038406 PMC7613579

[11] Radner JM Ferrer MJ McMahon D Shankar AH Silver KL. Practical considerations for transitioning early childhood interventions to scale: lessons from the saving brains portfolio. Ann N Y Acad Sci. (2018) 1419:230–48. doi: 10.1111/nyas.13684, PMID: 29791735 PMC9764263

[12] Cavallera V Tomlinson M Radner J Coetzee B Daelmans B Hughes R. Scaling early child development: what are the barriers and enablers? Arch Dis Child. (2019) 104:S43–50. doi: 10.1136/archdischild-2018-315425, PMID: 30885965 PMC6557300

[13] Durlak JA DuPre EP. Implementation matters: a review of research on the influence of implementation on program outcomes and the factors affecting implementation. Am J Community Psychol. (2008) 41:327–50. doi: 10.1007/s10464-008-9165-0, PMID: 18322790

[14] Nilsen P Bernhardsson S. Context matters in implementation science: a scoping review of determinant frameworks that describe contextual determinants for implementation outcomes. BMC Health Serv Res. (2019) 19:189. doi: 10.1186/s12913-019-4015-3, PMID: 30909897 PMC6432749

[15] Westgard C Fleming WO. The use of implementation science tools to design, implement, and monitor a community-based mHealth intervention for child health in the Amazon. Front Public Health. (2020) 8:411. doi: 10.3389/fpubh.2020.00411, PMID: 32974257 PMC7466738

[16] Krishna D Muthukaruppan SS Bharathwaj A Ponnusamy R Poomariappan BM Mariappan S. Rapid-cycle evaluation in an early intervention program for children with developmental disabilities in South India: optimizing service Providers' quality of work-life, family program engagement, and school enrollment. Front Public Health. (2020) 8:567907. doi: 10.3389/fpubh.2020.567907, PMID: 33330314 PMC7734086

[17] Olusanya BO Davis AC Wertlieb D Boo NY Nair MK Halpern R. Developmental disabilities among children younger than 5 years in 195 countries and territories, 1990-2016: a systematic analysis for the global burden of disease study 2016. Lancet Glob Health. (2018) 6:e1100–21. doi: 10.1016/S2214-109X(18)30309-7, PMID: 30172774 PMC6139259

[18] Ministry of Statistics and Program Implementation. Disabled persons in India: a statistical profile. (2016). Available at: https://ruralindiaonline.org/library/resource/disabled-persons-in-india-a-statistical-profile-2016

[19] Muthukaruppan SS Cameron C Campbell Z Krishna D Moineddin R Bharathwaj A. Impact of a family-centred early intervention programme in South India on caregivers of children with developmental delays. Disabil Rehabil. (2020):2410–9. doi: 10.1080/09638288.2020.1836046, PMID: 33103498

[20] World Health Organization (WHO). Community-based rehabilitation: CBR guidelines. Geneva: World Health Organization (2010). 26290927

[21] Lukersmith S Hartley S Kuipers P Madden R Llewellyn G Dune T. Community-based rehabilitation (CBR) monitoring and evaluation methods and tools: a literature review. Dis Rehabil. (2013) 35:1941–53. doi: 10.3109/09638288.2013.770078, PMID: 23574396

[22] United nations Children’s fund (UNICEF). Program Guidance: Strengthening shock responsive social protection systems. Social inclusion and policy, New York, NY, USA (2019). PD/GUIDANCE/2019/005. Available at: https://www.unicef.org/media/63846/file

[23] United Nations Educational Scientific and Cultural Organization (UNESCO). N for nose – state of the education report for India – children with disabilities. (2019). Available at: https://en.unesco.org/news/n-nose-state-education-report-india-2019-children-disabilities

[24] Krapels J van der Haar L Slemming W de Laat J Sanou AS Holding P. The aspirations of measurement for change. Front Public Heal. (2020) 8:234. doi: 10.3389/fpubh.2020.568677, PMID: 33330315 PMC7732529

[25] Van der Haar L Holding PA Krapels J de Laat J Slemming W. Measurement for change: from idea to approach. Front Public Health (2020) 8;:581756. doi: 10.3389/fpubh.2020.581756. PMID: ; PMCID: . 33330322 PMC7732670

[26] The World Bank. World Bank approves $162 million to strengthen social protection Systems for Persons with Disabilities in Tamil Nadu. [Press Release]. (2022). Available at: https://www.worldbank.org/en/news/press-release/2022/06/14/world-bank-approves-162-million-to-strengthen-social-protection-systems-for-persons-with-disabilities-in-tamil-nadu

[27] Msall ME DiGaudio K Duffy LC LaForest S Braun S Granger CV. WeeFIM. Normative sample of an instrument for tracking functional independence in children. Clin Pediatr (Phila). (1994) 33:431–8. doi: 10.1177/000992289403300709, PMID: 7955782

[28] Jegan A. Demand for higher education – a comparison between India and Tamil Nadu. Int J Creat Res Thoughts. (2020) 8:4448–69.

[29] Pattnaik S Murmu J Agrawal R Rehman T Kanungo S Pati S. Prevalence, pattern and determinants of disabilities in India: insights from NFHS-5 (2019–21). Front Public Health. (2023) 11:1036499. doi: 10.3389/fpubh.2023.1036499, PMID: 36923034 PMC10009251

[30] Saikia N Moradhvaj BJK. Gender difference in health-care expenditure: evidence from India human development survey. PLoS One. (2016) 11:e0158332. doi: 10.1371/journal.pone.0158332, PMID: 27391322 PMC4938214

[31] People Research on India’s Consumer Economy. (2021). Available at: https://www.price360.in/

[32] Milner KM Salazar RB Bhopal S Brentani A Britto PR Dua T. Contextual design choices and partnerships for scaling early child development programmes. Arch Dis Child. (2019) 104:S3–S12. doi: 10.1136/archdischild-2018-315433, PMID: 30885961 PMC6557220

[33] Magnusson D Sweeney F Landry M. Provision of rehabilitation services for children with disabilities living in low- and middle-income countries: a scoping review. Disabil Rehabil. (2019) 41:861–8. doi: 10.1080/09638288.2017.1411982, PMID: 29212379

[34] Shonkoff JP Radner JM Foote N. Expanding the evidence base to drive more productive early childhood investment. Lancet. (2017) 389:14–6. doi: 10.1016/S0140-6736(16)31702-0 27717609

[35] O'Cathain A Croot L Duncan E Rousseau N Sworn K Turner KM. Guidance on how to develop complex interventions to improve health and healthcare. BMJ Open. (2019) 9:e029954. doi: 10.1136/bmjopen-2019-029954, PMID: 31420394 PMC6701588

[36] Singh AK Kumar R Mishra CK Khera A Srivastava A. Moving from survival to healthy survival through child health screening and early intervention services under Rashtriya Bal Swasthya Karyakram (RBSK). Indian J Pediatr. (2015) 82:1012–8. doi: 10.1007/s12098-015-1823-2, PMID: 26199076

[37] Smythe T Zuurmond M Tann CJ Gladstone M Kuper H. Early intervention for children with developmental disabilities in low and middle-income countries – the case for action. Int Health. (2021) 13:222–31. doi: 10.1093/inthealth/ihaa044, PMID: 32780826 PMC8079317

