# Supplementary material for: Leveraging monitoring, evaluation, and learning to scale the Enabling Inclusion® program for children with disabilities in India and globally
Source: Front Public Health. 2023 Dec 12;11:1165034. doi: 10.3389/fpubh.2023.1165034 (PMC10757565; doi:10.3389/fpubh.2023.1165034)
Supplement: Supplementary file 1 [file Table_1.DOCX]

**Supplementary Material**

**Leveraging Monitoring, Evaluation, and Learning to Scale the Enabling Inclusion**® **Program for Children with Disabilities in India and Globally**

Marie Brien^*^, Franzina Coutinho, Dinesh Krishna, Lotte van der Haar, Joost de Laat, Sankara Raman Srinivasan, Navamani Venkatachalapathy

***Correspondence:** Marie Brien: [researchassociate@amarseva.org](mailto:researchassociate@amarseva.org)

**Table S1. EI Program Indicators Tracked**

**Service Providers**

Total number of people using the app

Type of providers:

Program Management and Field Team Leaders

Physiotherapists (PTs)

Occupational Therapists (OTs)

Special educators

Speech trainers/ Speech Therapists / Speech Language Pathologists

Community rehabilitation workers

            Developmental Paediatricians

Administrators

Information Technology managers

**Screening**

# of children screened

Age range and gender of children screened

% screened with Trivandrum Development Screening Chart (TDSC)

% screened with the UNICEF Child Functioning Module

% screened positive (requiring child rehab / early intervention services)

**Children and Families Served**

# of children receiving child rehab / early intervention services

# of caregivers empowered

# of children enrolled in school

# of children accessing government benefits (e.g. disability cards, grants)

# of parents participating in parent groups / self help-groups

# of assistive devices provided to children

**Awareness Programs**

# of people attending awareness programs

Age and gender of attendees

Knowledge, Attitudes and Practices pre and post training surveys

**Training Programs**

# of people attending training programs

Age range and gender of attendees

Types of workers (community, health, preschool, school)

Knowledge, Attitudes and Practices pre and post training surveys

**Child Demographic Characteristics**

Age at enrolment

Gender

Enrolled in school at baseline

Type of school

**Parent Demographic Characteristics**

Primary caregiver

Age of primary caregiver

Religion

Education

Employment

Yearly Income

Below poverty line (Yes / No)

**Distribution of Child Impairments in the EI ® Program**

Child Impairments (% of children)

Speech

Cognitive

Physical

Behavioural

Hearing

Vision

Child Primary Impairment (% of children)

Speech

Cognitive

Physical

Behavioural

Hearing

Disability Diagnosis Category (n)%

Disability Sub classification (n)%

**Types of EI® App Intervention Provided**

Physiotherapy Interventions (% of children)

*Subclassifications:*

         Cerebral Palsy

         Muscular Dystrophy

         Spina Bifida

         Spinal Deformity

         CTEV - Clubfoot

         Equinus deformity

         Erb's Palsy

         Torticollis (Wry Neck)

         Gross Motor

         Fine Motor

Special Education Interventions (% of children)

*Subclassifications:*

         Intellectual Disability

         Autism

         Cerebral Palsy & Multiple Disability

         Attention Deficit Hyperactivity Disorder

         Low Vision

         Total Blindness

         Deaf and Blind

Speech, Language and Communication Interventions (% of children**)**

Environmental Interventions (% of children**)**

Prevocational Interventions (% of children**)**

Vocational Interventions (% of children**)**

Inclusion Interventions (% of children**)**

**Standardized Child Outcomes Measures**

GMFM (Gross Motor Function Measure) which measures gross motor function.

FACP (Functional Assessment Checklist for Programming) which measures personal, social, academic, occupational and recreational domains of development

MDPS (Madras Developmental Programming System Behavioral Scale) which measures behaviors and milestones across cognitive, social, emotional, and motor skills.

WEE-FIM (Pediatric Functional Independence Measure) which measures cognition, self-care, mobility and communication.

Additional impairment specific evaluations

**Family Outcome Measures**

COPM (Canadian Occupational Performance Measure) – help parents set therapeutic goals for their child and measures parents’ satisfaction and performance of achieving these goals.

CI (Caregiver Child Interaction) - measures interaction between caregivers and their children.

MCSI (Modified Caregiver Strain Index) - measures strain / stress in caregivers of children with delayed development.

FES (Family Empowerment Scale) - measures empowerment in caregivers of children with delayed development.

Parent Program Evaluation Survey

**Table S2. Key Performance Indicators (KPI) for Completion Rate of EI® app Modules**

| **EI app Module** | **Description of KPI** |
| --- | --- |
| Screening Module | Percentage of children screened positive / identified with developmental delay that are converted into active service users within the program. |
| Service User Module | Percentage of children with active intervention / treatment plans that are ongoing |
| Assessment module | Percentage of children with initial assessments completed (data is broken down by different types of initial assessments) |
| Family-Centered Care Module | Percentage of caregivers with their feedback forms, goal-setting forms, caregiver strain and empowerment indexes completed. |
| Evaluation module | Percentage of children with their developmental score evaluations completed (data is broken down by different types of domain specific evaluations) |
| Scheduling / Daily activity module | Percentage of planned service provider therapy and non-therapy activities completed |
